# Endocrine profile and OPU-ICSI outcomes in mares: a comparative study

**DOI:** 10.1530/RAF-25-0027

**Published:** 2025-07-04

**Authors:** Dragos Scarlet, Gerhard Schuler, Eleni Malama, Heinrich Bollwein, Carlotta Bocci, Silvia Colleoni, Giovanna Lazzari, Cesare Galli, Mariusz Pawel Kowalewski

**Affiliations:** ^1^ Institute of Veterinary Anatomy, Vetsuisse Faculty Zurich, Zurich, Switzerland; ^2^ Clinic of Reproductive Medicine, Vetsuisse Faculty Zurich, Zurich, Switzerland; ^3^ AgroVet-Strickhof, Vetsuisse Faculty, Lindau, Switzerland; ^4^ Veterinary Clinic for Reproductive Medicine and Neonatology, Justus-Liebig-University, Giessen, Germany; ^5^ Avantea, Cremona, Italy

**Keywords:** OPU-ICSI outcome, horses, OSFs, progesterone

## Abstract

**Abstract:**

Despite recent improvements in equine ovum pick-up (OPU) combined with intracytoplasmic sperm injection (ICSI), there is still significant inter-individual variability. In this study, serum concentrations of the oocyte-secreted factors growth differentiation factor 9 (GDF9) and bone morphogenetic protein 15 (BMP15), as well as the GDF9/BMP15 complex, anti-Müllerian hormone (AMH), progesterone (P4), and free and conjugated estradiol-17β (E2), were determined in 59 mares (3–24 years) at the time of OPU. Mares were classified retrospectively, based on the number of embryos obtained, into low or high outcome groups, using two or three embryos as the threshold values. Results of OPU-ICSI were not affected by the mare’s age, body condition score, cycle stage, number of aspirated follicles, number of recovered oocytes, or the recovery rate (*P* > 0.05 in all cases). Mares with <2 or ≥2 embryos had similar hormone levels (*P* > 0.05 in all cases), but there was a >90% likelihood of obtaining ≥2 embryos per session when P4 and E2 concentrations were considered simultaneously. Mares with ≥3 embryos/session had lower P4 and higher AMH (*P* < 0.05 in both cases). GDF9 and BMP15 were detectable in 14 and 12% of mares, respectively, while the GDF9/BMP15 complex was detected in 93% of mares, with no correlation to other factors. In conclusion, these preliminary findings suggest a negative correlation between high P4 levels and OPU-ICSI outcome in horses.

**Lay summary:**

We analyzed the relationship between the hormone profiles of mares at the time of egg collection and the outcome of intracytoplasmic sperm injection (ICSI), as ICSI success often varies significantly between individuals. There were no differences in the hormone profiles of mares producing fewer than two or two or more embryos. However, mares with three or more embryos/session showed significant differences in their hormone profiles compared to those producing fewer than three embryos/session. These changes were mainly related to steroid hormones produced by the ovary, such as progesterone and estradiol, and to the concentration of AMH. Our results suggest a possible influence of the hormone profile of the mare on the number of embryos obtained.

## Introduction

Ovum pick-up (OPU) combined with intracytoplasmic sperm injection (ICSI) is used routinely to obtain foals from mares that are genetically valuable or have poor fertility. Equine OPU-ICSI can be performed throughout the year with similar results not only in breeding centers but also at remote locations as an outpatient procedure (Galli *et al.* 2016*a*, Fonte *et al.* 2024). With recent improvements, 60–70% of the oocytes obtained reach metaphase II (MII) after *in vitro* maturation (IVM) (Papas *et al.* 2021, Fonte *et al.* 2024). Subsequently, 20–30% of these MII oocytes develop into a blastocyst after ICSI (Lazzari *et al.* 2020, Fonte *et al.* 2024). The low blastocyst rates obtained from oocytes undergoing IVM, <20% (Lazzari *et al.* 2020), are approximately half of those achieved in cattle (Ferré *et al.* 2023). However, as there is often a large inter-individual variability in outcome, the mare herself is the most reliable predictor of success (Cuervo-Arango *et al.* 2019*b*), although the male factor should not be completely ignored (Galli *et al.* 2016*b*).

When serum concentrations of anti-Müllerian hormone (AMH, a member of the transforming growth factor beta (TGF-β) superfamily) were determined in an attempt to predict equine OPU-ICSI outcome, the authors concluded that while AMH concentrations could help identify mares with higher chances of producing multiple embryos, it should not be used as a sole predictor due to the significant inter-individual variability (Papas *et al.* 2021). Nonetheless, in different studies, AMH was positively correlated with the number of antral follicles (Claes *et al.* 2015, Scarlet *et al.* 2018). In addition, AMH showed high long-term consistency within individual mares, and its concentration was not influenced by cycle stage or pregnancy (Claes *et al.* 2015). However, it is important to note that AMH, which is produced by the granulosa cells of pre-antral and antral follicles, serves as a reliable marker of ovarian reserve, but it does not indicate oocyte quality in women (Buratini *et al.* 2022). In this context, growth differentiation factor 9 (GDF9) and bone morphogenetic protein 15 (BMP15), which belong to the TGF-β superfamily and are secreted solely by the oocyte in several species such as rabbits, mice, sheep, and cattle (El-Fouly *et al.* 1970, Dong *et al.* 1996, Galloway *et al.* 2000, Juengel *et al.* 2009), may provide a more accurate reflection of oocyte quality and/or ovarian function. Several mutations in GDF9 or BMP15 genes have been identified as the causal mechanism underlying the highly prolific or infertile nature of various sheep strains (Otsuka *et al.* 2011). In humans, the expression of *GDF9* and *BMP15* in cumulus cells is closely associated with oocyte maturation, fertilization, embryo quality, and pregnancy outcome (Li *et al.* 2014), whereas in horses, their expression in oocytes is associated with oocyte development (Scarlet *et al.* 2017) and age of the mare (Campos-Chillon *et al.* 2015). Moreover, GDF9 and BMP15 are released into the follicular fluid where they act via paracrine signals to regulate proliferation, metabolism, and differentiation of granulosa cells (Gilchrist *et al.* 2008).

Steroid hormones, the primary endocrine products of granulosa cells, also play a crucial role in regulating follicular growth and oocyte differentiation. The horse is peculiar among domestic animals, as significant amounts of sulfoconjugated estrogens are produced in the preovulatory follicle (Brown *et al.* 2006), and positive effects of estrogen inactivation on equine embryo production have been suggested (Metcalfa *et al.* 2017, Fonte *et al.* 2024). Interestingly, the total serum estrogens (conjugated and unconjugated) are present in 100-fold higher concentrations than estradiol during the follicular phase in mares (Palmer & Terqui 1977). While estrone sulfate concentrations have been well described (Makawiti *et al.* 1983, Koskinen *et al.* 1989), estradiol sulfate has only been reported in the serum of pregnant mares (Legacki *et al.* 2019). In our recently published study, there was a negative correlation between intrafollicular BMP15 and estradiol sulfate concentrations (Samie *et al.* 2025).

Besides their intrafollicular paracrine/autocrine functions, both GDF9 and BMP15 have also been detected in the serum of women, with no significant changes in their concentrations observed across the menstrual cycle and no correlation with AMH or baseline FSH levels (Riepsamen *et al.* 2019). Furthermore, serum BMP15 and GDF9 concentrations are not affected in patients with polycystic ovary syndrome (Riepsamen *et al.* 2023) or endometriosis (Shamsa *et al.* 2023). However, serum BMP15 levels are reduced in obese women, though the functional implications remain unclear (Arıkan & Sagsoz 2023). Following the recent validation of human ELISA assays for equine follicular fluid, we aimed to analyze the relationship between serum concentrations of GDF9, BMP15, and the GDF9/BMP15 complex at the time of oocyte collection and OPU-ICSI outcomes, as well as their association with steroid hormones in mares. This study marks an initial step in evaluating whether these oocyte-secreted growth factors could serve as potential biomarkers of oocyte quality and/or ovarian function. Concomitantly, other ovarian products such as progesterone, estradiol-17β, 17β-estradiol-3-sulfate, and AMH were investigated, as there is currently no information about their relationship with the oocyte-secreted factors GDF9 and BMP15, or about their relationship with OPU-ICSI outcomes besides AMH in horses.

## Materials and methods

### Horses

Samples used in this study were collected from animals undergoing commercial *in vitro* embryo production requested by the animal owners as part of reproductive management, and no extra manipulation was performed for the purpose of the study; therefore, no ethical approval was required. A total of 59 mares (Warmblood: *n* = 48, Quarter Horse: *n* = 11) between 3 and 24 years of age and with a body condition score (BCS) between 4 and 8 (Henneke *et al.* 1983) that underwent OPU-ICSI at the AVANTEA clinic (Italy) during November 2023 were included in this study. Based on ultrasonographic examination of the genital tract and peripheral progesterone concentrations, 19 mares were in estrus (32%), 30 in diestrus (51%), and ten in transition phase (17%) at the time of oocyte collection. The mares were in good general health condition, as this is a prerequisite for acceptance in a clinical OPU-ICSI program. They were housed in individual boxes and fed with 8 kg hay twice a day and 2 kg alfalfa pellets in the evening. Mares with a BCS below 5 were given 2 kg of cereals in the morning. All mares had daily access to the paddock for a few hours if the weather allowed it. Only one OPU-ICSI session per mare was included in this study. Based on the published average of 1–2 embryos/session (Papas *et al.* 2021, Claes & Stout 2022, Fonte *et al.* 2024), mares were retrospectively classified into low or high outcome groups based on the number of blastocysts obtained. Statistical analyses were performed for groups based on thresholds of two and three embryos per OPU-ICSI session, respectively. As there was an even distribution of mares from each breed in the outcome groups, this variable did not affect any of the subsequently analyzed parameters.

### Ovum pick-up

Oocyte collection was performed as previously described (Lazzari *et al.* 2020). Briefly, the donor mares were transrectally ultrasound scanned 1–3 days before the OPU day to assess the number and size of ovarian follicles. The procedure was performed if at least eight to ten follicles of 1 cm diameter were present. For OPU preparation, mares were sedated with detomidine hydrochloride (4 mg, administered three times or more as needed for adequate sedation) and given an epidural anesthesia (4–8 mL of 2% lidocaine). Antral follicles were punctured using a 12G double-lumen needle mounted on a modified native endovaginal probe and flushed eight to ten times with Euroflush media (IMV, France) containing 5 IU/mL of heparin (Heparin-Natrium 5,000 UI/mL, B.Braun Melsungen AG, Germany) to prevent clotting. The recovered flushing fluid was passed through an embryo filter (EZ Way Filter, USA), and the oocytes were collected from the fluid remaining in the filter. Oocytes were washed twice in HEPES-buffered synthetic oviductal fluid (H-SOF), photographed, and then transferred to maturation media. If necessary, they were held at ambient temperature to synchronize maturation with other batches according to laboratory scheduling.

### IVM, ICSI, and *in vitro* culture (IVC)

After collection, immature oocytes were kept in holding media at 22–24°C for 2–12 h before being transferred to maturation media for 36–38 h at 38°C under 5% CO2 in air, as previously described (Lazzari *et al.* 2020). The IVM medium was a 1:1 mixture of Ham’s F12 and Dulbecco’s Modified Eagle’s Medium, containing 0.1 IU/mL human follicle-stimulating hormone, 0.1 IU/mL luteinizing hormone (Menopur, Ferring, Belgium), epidermal growth factor (50 ng/mL, PeproTech, Thermo Fisher, Italy), 1 mM sodium pyruvate, 10% fetal calf serum (Gibco, Thermo Fisher), and insulin-transferrin-selenium supplement. Following IVM, oocytes were transferred to H-SOF supplemented with 2.5 mg/mL hyaluronidase for 4–8 min, followed by cumulus cell removal by pipetting. Consequently, denuded oocytes were returned to the IVM medium until ICSI.

Frozen stallion semen was thawed at ambient temperature and prepared using density gradient centrifugation (45 and 90% RediGrad, Amersham Biosciences AB, Sweden) for 20 min at 700 *g*, followed by a 5 min wash in Ca-free TALP at 400 *g*. Post-centrifugation, the semen pellet was resuspended in a modified SOF-IVF medium as described in (Lazzari *et al.* 2002), containing sperm motility solution (20 μM penicillamine, 100 μM hypotaurine, and 1 μM epinephrine) and incubated at 38°C. Just before ICSI, the semen suspension was diluted 1:1 with 10% polyvinylpyrrolidone (PVP 360) in H-SOF and dispensed in 3 μL drops covered with mineral oil. ICSI was performed on metaphase II oocytes using a Nikon inverted microscope with a heated plate (37.5°C), a Narishige micromanipulator, and an Eppendorf PiezoXpert (intensity 10–30, speed 10, infinite pulse).

After ICSI, injected metaphase II oocytes were incubated at 38°C in a humidified atmosphere containing 5% CO2 and 5% O2 and cultured in a modified SOF-IVC medium with bovine serum albumin (BSA) and amino acids, as previously described (Lazzari *et al.* 2002). Cleavage was assessed 2 days after ICSI, with non-cleaved embryos being removed. On day 6, approximately half of the modified SOF-IVC medium was replaced with the same medium supplemented with 10% of a 1:1 mixture of fetal calf serum and KnockOut Serum Replacement (Thermo Fisher). Blastocyst formation was assessed twice daily from day 6 to day 9 (day 0 being the day of ICSI).

Unless otherwise stated, all reagents were purchased from Sigma-Aldrich (Switzerland).

### Hormone assays

At the start of the OPU procedure, blood samples were collected from the jugular vein using sampling tubes without separating gel, and serum was collected after centrifugation (2,000 *g*, 10 min at 4°C) and stored at −20°C until further analysis.

The radioimmunoassay (RIA) measurement of progesterone (P4) concentrations was carried out as previously described and applied in several animal species, including the horse (Hoffmann *et al.* 1973, Hoffmann *et al.* 1996). Before radioimmunological determination, 50 μL serum was extracted twice with hexane. The pooled extracts were dried in a vacuum evaporator (MicroDancer, Hettich AG, Switzerland) and re-dissolved in assay buffer. The cross-reactivity of the antiserum with other steroids is as follows: androstenedione <0.01%, DHEA <0.01%, estradiol <0.01%, estrone <0.01%, cortisol <0.01%, testosterone 0.37%, 17α-hydroxy-pregnenolone <0.01%, 17α-hydroxy-progesterone 0.49%, and pregnenolone 0.69%. The lower limit of detection was 0.25 ng/mL, and the intra- and interassay coefficients of variation were 8.8 and 8.9%, respectively.

Estradiol-17β (E2) was measured using a sensitive RIA, as previously described (Hoffmann *et al.* 1992, Klein *et al.* 2003). The serum samples (0.2 mL) were extracted twice with toluene, and the dried and re-dissolved extracts were subjected to radioimmunological determination using an antiserum highly specific for E2. The lower limit of detection was 2.5 pg/mL, and the intra- and interassay coefficients of variation were 7.1 and 12.0%, respectively. The same RIA was used for the determination of 17β-estradiol-3-sulfate (E2S). For this purpose, the aqueous sample residues remaining after extraction of estradiol were enzymatically hydrolyzed (β-glucuronidase/arylsulfatase from Helix pomatia – Roche Diagnostics GmbH, Mannheim, Germany), re-extracted, and then the concentration of the conjugated form was determined as described for E2.

Serum AMH concentrations were determined using an equine-specific ELISA assay (AL-115, Ansh Labs, USA), as previously described (Scarlet *et al.* 2018), according to the manufacturer’s instructions. The intraassay and interassay coefficients of variation were less than 10%, and the minimal detectable concentration was 0.07 ng/mL.

For GDF9 and BMP15 assessment, ELISA assays established for the measurement of the human molecules (AL-176 and AL-179, Ansh Labs) and recently validated for equine follicular fluid in our laboratory (Samie *et al.* 2025), were used. In both cases, the intraassay and interassay coefficients of variation were less than 10%, and the minimal detectable concentration was 20 pg/mL for GDF9 and 50 pg/mL for BMP15.

The serum concentrations of the GDF9/BMP15 complex were quantified using an ELISA (AL-181, Ansh Labs, USA) established for use with human serum. Serial dilutions of equine serum showed good parallelism to the standard curve (deviation within 10% of expected values). As per the manufacturer`s recommendations, 25 μL undiluted serum was used. The intraassay and interassay coefficients of variation were 6.7 and 11.8%, respectively, and the minimal detectable concentration was 20 arbitrary units (AU) per mL.

### Statistical analysis

The R language and environment for statistical computing version 4.2.2 was used for data analysis. The distribution of each continuous variable was checked for deviations from normality by visual inspection (histogram, quantile–quantile plots), and by performing the Shapiro-Wilk test. Differences in continuous variables between embryo groups were assessed with the Mann–Whitney U test at the 0.05 significance level. The Spearman’s rank correlation coefficient *r_s_* was computed to describe the correlation between continuous variables at the 0.05 significance level. Principal component analysis was performed to detect any potential underlying dimensions in the data of the mare’s hormonal profile; the number of follicles and oocytes, and the blastocyst rate were included as supplementary quantitative variables.

The relation of the response variables (embryo group) with a mare’s hormonal profile and other mare-dependent factors (age, breed, BCS, cycle stage) was assessed by means of generalized linear mixed models, which incorporate both fixed effects (independent variables) and random effects (mare) in a linear predictor, via maximum likelihood. The linear predictor was related to the conditional mean of the response through the logit link function of the binomial family. Model parameters were estimated using the method of maximum likelihood. Generalized linear mixed-effects modeling was performed using the glmer function of the lme4 statistical package. The distribution of model parameter values was estimated using *n* = 500 bootstrap samples and fitting the models for the resampled data. The Spearman’s rank correlation coefficient (*r_s_*) was computed to describe the relation between continuous variables (at the 0.05 significance level).

The random forests (RF) supervised learning method was performed to identify predictors that would be important for the assignment of individuals to one of the OPU-outcome groups. The RF ensemble uses different bootstrap samples of the original data to construct a set of *n* = ntree decision trees. Each decision tree is a flowchart-like model with the aim to reduce the variance of the outcome variable after each branching point (node). At each node, one predicting variable (hormone) is selected as the optimal classifier (the one that results in the highest possible reduction of data variance) out of a sample of variables. For each tree, the % error rate of the out-of-bag (OOB) sample data is recorded. The mean OOB error is a method of measuring the prediction error of RF using bootstrap aggregating (bagging) to sub-sample data used for training. The importance of each predictor is evaluated through two metrics: the mean decrease in accuracy (MDA), and the mean decrease in Gini index (MDGI). The MDA of each predicting variable expresses the increase in the OOB error rate after randomly permuting the values of the variable; the higher the MDA value of a variable, the more important the variable for the classification of samples. The Gini index is a measure of how each variable contributes to the purity of the nodes of the decision trees. Each time a particular variable is used to split a node, the Gini index for the child nodes is calculated and compared to that of the parent node; variables that result in nodes with higher purity have a higher MDGI value and are considered more important for the classification of samples. In this dataset, we grew *ntree* = 500 trees for each classifier, with three variables randomly sampled as candidate predictors at each node split.

## Results

### General observations

From the 59 mares and cycles included in the study, 803 oocytes were aspirated by OPU from 1,111 follicles (72.3% recovery rate), 526 of which reached the metaphase II stage, with a mean maturation rate of 68.2%. All the mature oocytes were fertilized by ICSI, and 457 cleaved (86.6% mean cleavage rate), resulting in 127 blastocysts after IVC. The blastocyst rate, calculated as a function of the total number of recovered oocytes, was 15.8%, with an average of 2.2 embryos/session (range 0–9). Specifically, there was no embryo produced in 10/59 cases (16.9%), one embryo in 15/59 cases (25.4%), two embryos in 11/59 cases (18.6%), three embryos in 13/59 cases (22.0%), and more than three embryos in 10/59 cases (16.9%). All embryos included in this study were grade 1, while low-grade and late embryos were not considered. Significant positive correlations were observed between the number of aspirated follicles and the number of oocytes collected (*r_s_* = 0.78, adjusted *P* < 0.001), as well as the AMH levels (*r_s_* = 0.41, adjusted *P* = 0.001). As expected, there was a positive correlation between the number of blastocysts and the blastocyst rate (*r_s_* = 0.86, adjusted *P* < 0.001), and the E2 and E2S concentrations were positively correlated with each other (*r_s_* = 0.56, adjusted *P* < 0.001).

### Serum GDF9, BMP15, and GDF9/BMP15 complex concentrations

This was the first time that concentrations of the oocyte-secreted GDF9 and BMP15 homodimers, as well as the GDF9/BMP15 heterodimer, were determined in equine serum. We could only find GDF9 in 8/59 (14%) samples and BMP15 in 7/59 (12%) samples, respectively (Table 1). Of the OPU-ICSI outcome and endocrine characteristics assessed relative to GDF9 and BMP15, there were no significant differences between undetectable and detectable GDF9 and BMP15 groups, except for the GDF9/BMP15 heterodimer relative to GDF9 (*P* < 0.05, Table 1). Of the 59 mares included in the study, only two (4%) had both GDF9 and BMP15 serum levels above the detection limits of the assays. The GDF9/BMP15 complex was detectable in 55/59 (93%) of the samples, and its serum levels did not differ among groups based on the number of embryos produced per OPU-ICSI cycle. Neither GDF9 or BMP15 homodimer, nor GDF9/BMP15 heterodimer, showed a correlation with any of the other factors.

**Table 1:** OPU-ICSI outcome and endocrine characteristics comparing mares with detectable and undetectable serum concentrations of GDF9 and BMP15. Data are mean (95% CI), with comparisons of detectable and undetectable groups analyzed by Mann–Whitney U test. A *P* value <0.05 was considered significant (highlighted in bold).

Characteristics		GDF9		*P*	BMP15		*P*
	All mares	Detectable (≥20 pg/mL)	Undetectable (<20 pg/mL)		Detectable (≥50 pg/mL)	Undetectable (<50 pg/mL)	
*n*	59	8	51		7	52	
Age (years)	13.3 (11.8–14.8)	15.8 (10.9–20.6)	12.9 (11.3–14.6)	0.202	11.6 (4.4–18.8)	13.5 (12.0–15.1)	0.621
BCS	5.5 (5.3–5.8)	5.9 (4.8–6.9)	5.5 (5.3–5.7)	0.345	5.4 (4.9–5.9)	5.6 (5.3–5.8)	0.828
Aspirated follicles	18.8 (16.6–21.0)	15.6 (10.1–21.1)	19.3 (16.9–21.7)	0.194	17.3 (12.1–22.5)	19.0 (16.6–21.5)	0.973
Recovered oocytes	13.6 (11.9–15.3)	12.1 (8.7–15.6)	13.8 (12.0–15.3)	0.681	13.6 (10.3–16.8)	13.6 (11.7–15.5)	0.473
Recovery rate (%)	73.5 (69.4–77.7)	80.5 (68.5–92.6)	72.4 (67.9–76.9)	0.188	81.4 (66.5–96.2)	72.5 (68.1–76.8)	0.214
Maturation rate (%)	67.1 (63.3–70.8)	74.6 (62.0–87.3)	65.9 (61.9–69.9)	0.156	66.1 (54.4–77.8)	67.2 (63.1–71.3)	0.860
Cleavage rate (%)	86.6 (82.3–90.3)	77.6 (60.2–94.9)	88.0 (84.5–91.5)	0.052	88.4 (75.3–101.5)	86.3 (82.4–90.3)	0.724
Blastocysts	2.2 (1.7–2.6)	1.9 (0.4–3.4)	2.2 (1.7–2.7)	0.726	2.0 (0.5–3.5)	2.2 (1.7–2.7)	1.000
Blastocyst rate (%)	17.4 (13.9–21.0)	18.7 (3.1–34.2)	17.2 (13.7–20.8)	0.965	15.9 (2.8–29.1)	17.6 (13.9–21.4)	0.805
P4 (ng/mL)	5.1 (3.7–6.5)	4.2 (−0.6 to 9.1)	5.2 (3.7–6.8)	0.379	4.9 (−2.9 to 12.6)	5.1 (3.7–6.5)	0.375
E2 (pg/mL)	7.8 (6.9–8.7)	7.1 (5.8–8.4)	7.9 (6.9–9.0)	0.572	6.9 (4.3–9.5)	7.9 (7.0–8.9)	0.497
E2S (pg/mL)	90.6 (70.8–110.4)	84.3 (52.1–116.5)	91.6 (68.9–114.2)	0.492	114.9 (49.5–180.4)	87.3 (66.0–108.6)	0.252
AMH (pg/mL)	2,064.8 (1,592.2–2,537.4)	1,826.6 (884.3–2,768.8)	2,102.2 (1,566.6–2,637.8)	0.740	2,248.6 (1,123.4–3,373.8)	2,040.5 (1,514.7–2,565.4)	0.224
GDF9/BMP15 (AU)	644.8 (−59.2 to 1,348.9)	3,174.8 (−2,790.6 to 9,140.2)	248.0 (137.5–358.5)	**0.025**	3,520.3 (−3,543.4 to 10,584.1)	257.8 (149.8–365.7)	0.810

AU, arbitrary units; BCS, body condition score; P4, progesterone; E2, estradiol-17β; E2S, 17β-estradiol-3-sulfate; AMH, anti-Müllerian hormone; GDF9, growth differentiation factor 9; BMP15, bone morphogenetic protein 15.

### Characteristics of mares with OPU-ICSI outcome <2 or ≥2 embryos

**Figure 1:**
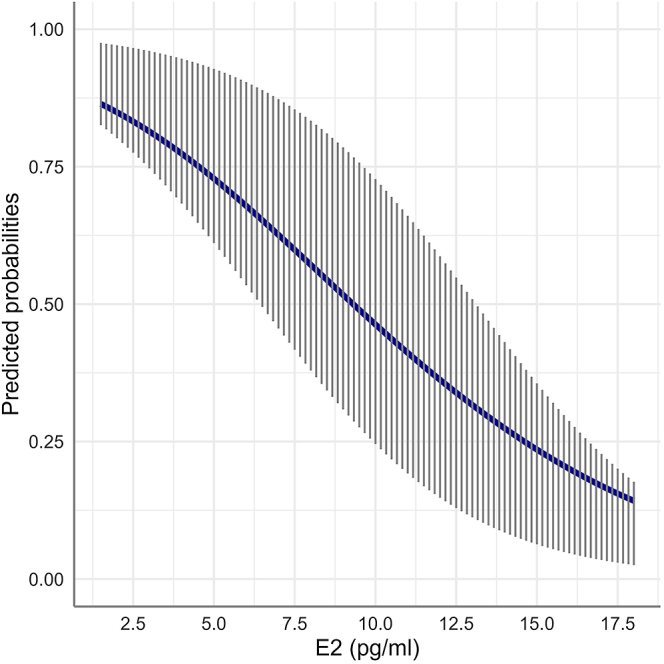
Average marginal probabilities (with the respective interquartile range) to obtain ≥2 embryos per OPU session (vs <2 embryos per OPU session) in relation to increasing serum E2 concentrations. Probabilities were computed after fitting generalized linear mixed models to 500 bootstrap samples of an original dataset of *n* = 59 mares and averaged across mares.

As presented in Table 2, there was no significant difference in the age, BCS, number of aspirated follicles, number of recovered oocytes, or recovery rate between the high and low OPU-ICSI outcome groups (*P* > 0.05 in all cases). In addition, there was no effect of the cycle stage on the number of embryos produced (*P* > 0.05). Despite similar numbers of oocytes being collected from both groups, the maturation, cleavage, and blastocyst rates were significantly higher in the group producing at least two blastocysts per session (*P* < 0.001). There was no difference in the endocrine profiles (P4, E2, E2S, AMH, GDF9, BMP15, and GDF9/BMP15 complex concentrations, respectively; *P* > 0.05 in all cases) of the mares, based on the threshold of two embryos/session. According to the generalized linear mixed model describing the relationship of the outcome of an OPU session (production of <2 vs ≥2 embryos per OPU session) with the mare’s hormonal profile, E2 concentration was the only deciding predictor of the dichotomous response variable. An increase in E2 concentration by 1 pg/mL (when all other predictors were kept constant) was linked to a 27% lower probability (odds ratio = 0.73, *P* = 0.012) of obtaining ≥2 embryos per OPU session (Fig. 1).

**Table 2:** Characteristics of mares assigned to the low versus high outcome groups based on the threshold of two embryos/session. Data are mean (95% CI), with comparisons of detectable and undetectable groups analyzed by Mann–Whitney U test. Different superscripts within a row indicate significant differences (*P* < 0.05, highlighted in bold).

Characteristics	OPU-ICSI outcome		*P*
	<2 blastocysts	≥2 blastocysts	
*n*	25	34	
Age (years)	12.3 (9.8–14.8)	14.1 (12.1–16.0)	0.196
BCS	5.4 (5.0–5.8)	5.6 (5.4–5.9)	0.196
Aspirated follicles	18.4 (15.0–21.9)	19.1 (16.1–22.1)	0.628
Recovered oocytes	13.4 (10.7–16.2)	13.7 (11.5–16.0)	0.717
Recovery rate (%)	74.5 (66.9–82.2)	72.8 (67.9–77.6)	0.369
Maturation rate (%)	62.1 (57.2–66.9)^a^	70.7 (65.4–76.1)^b^	**0.028**
Cleavage rate (%)	81.0 (73.9–88.2)^a^	90.7 (87.3–94.0)^b^	**0.036**
Blastocysts	0.6 (0.4–0.8)^a^	3.3 (2.8–3.8)^b^	**<0.001**
Blastocyst rate (%)	5.5 (3.0–8.0)^a^	26.2 (22.7–29.8)^b^	**<0.001**
P4 (ng/mL)	5.9 (3.5–8.3)	4.5 (2.7–6.3)	0.298
E2 (pg/mL)	8.9 (7.2–10.5)	7.0 (6.1–8.0)	0.123
E2S (pg/mL)	84.8 (60.4–109.1)	94.9 (64.6–125.2)	0.963
AMH (pg/mL)	1,761.1 (1,222.6–2,299.6)	2,288.1 (1,554.5–3,021.8)	0.510
GDF9/BMP15 (AU)	187.4 (138.4–237.0)	980.3 (−254.8 to 2,216.7)	0.624
GDF9 (pg/mL)	54.3 (7.8–100.8); (*n* = 4)	1,363.2 (−2,849.7 to 5,576.0); (*n* = 4)	0.886
BMP15 (pg/mL)	79.6 (6.1–153.0); (*n* = 3)	214.7 (−220.9 to 650.3); (*n* = 4)	0.629

AU, arbitrary units; BCS, body condition score; P4, progesterone; E2, estradiol-17β; E2S, 17β-estradiol-3-sulfate; AMH, anti-Müllerian hormone; GDF9, growth differentiation factor 9; BMP15, bone morphogenetic protein 15.

**Figure 2:**
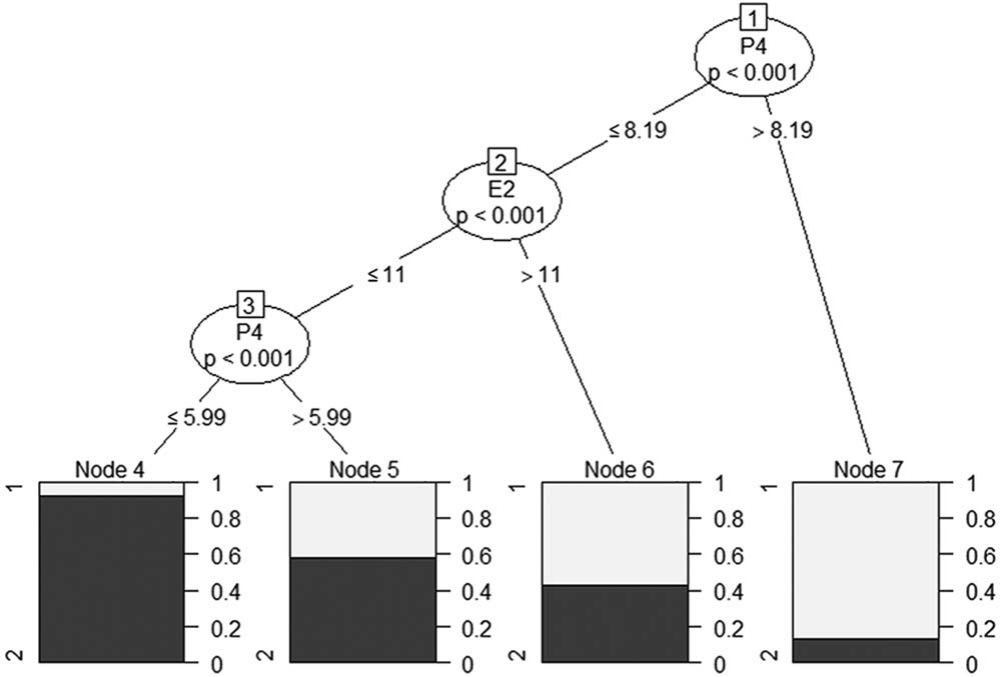
Example of RF tree with two continuous variables (P4, E2) as predictors of the embryo yield (production of ≥2 embryos vs <2 embryos per OPU session). Tree is grown with P4 as starting node. The cut-off value of the predictor is given along the branch (black line) originating from each decision node. Within each decision node, the predicted class probability *P* is also presented. At each terminal node, which no longer splits into sub-nodes, the ratio of mares producing ≥2 embryos (black area) or <2 embryos (gray area) is presented in a cumulative bar.

The levels of E2 made the most important contribution to the homogeneity of the nodes and leaves in the RF trees, as it was the independent variable with the highest MDGI. Moreover, E2 and P4 were the two variables with the highest MDA index. The RF had an OOB error rate of 40.7% overall; the error rate for predicting <2 embryos/OPU was 60%, while the prediction error for predicting ≥2 embryos was 26.5%. An example of RF decision trees built for the prediction of the dichotomous ‘embryo group 2’ variable is presented in Fig. 2. Particularly, mares with P4 ≤ 5.99 ng/mL (and simultaneously E2 ≤ 11.0 pg/mL) had a 92% likelihood of yielding ≥2 embryos. For mares with P4 levels in the range of 5.99–8.19 ng/mL, the chances of producing ≥2 embryos were 58% if E2 ≤ 11.0 pg/mL and 43% if E2 > 11.0 pg/mL, respectively. However, if P4 > 8.19 ng/mL, the expected OPU-ICSI yield per session was <2 embryos in 87% of the cases.

### Characteristics of mares with OPU-ICSI outcome <3 or ≥3 embryos

As presented in Table 3, there was no significant difference in the age, BCS, number of aspirated follicles, number of recovered oocytes, or the recovery rate between the high and low OPU-ICSI outcome groups (*P* > 0.05 in all cases). In addition, there was no effect of the cycle stage on the number of embryos produced (*P* > 0.05). Despite similar numbers of oocytes being collected from both groups, the maturation and blastocyst rates were significantly higher in the group producing at least three blastocysts per session (*P* < 0.001). There were no differences in the serum concentrations of E2, E2S, GDF9, BMP15, or GDF9/BMP15 complex, based on the threshold of three embryos/session (*P* > 0.05 in all cases). However, the mares that produced ≥3 embryos per session had lower P4 and higher AMH serum concentrations (*P* < 0.05 in both cases).

**Table 3:** Characteristics of mares assigned to the low versus high outcome groups based on the threshold of three embryos/session. Data are mean (95% CI), with comparisons of detectable and undetectable groups analyzed by Mann–Whitney U test. Different superscripts within a row indicate significant differences (*P* < 0.05, highlighted in bold).

Characteristics	OPU-ICSI outcome		*P*
	<3 blastocysts	≥3 blastocysts	
*n*	36	23	
Age (years)	13.4 (11.3–15.4)	13.2 (10.7–15.7)	1.000
BCS	5.5 (5.2–5.8)	5.6 (5.2–6.0)	0.615
Aspirated follicles	18.3 (15.3–21.3)	19.7 (16.4–23.0)	0.272
Recovered oocytes	13.1 (10.8–15.5)	14.3 (11.9–16.8)	0.145
Recovery rate (%)	72.8 (67.1–78.5)	74.7 (68.4–80.9)	0.786
Maturation rate (%)	63.8 (59.1–68.4)^a^	72.2 (66.1–78.4)^b^	**0.046**
Cleavage rate (%)	83.7 (78.3–89.1)	91.1 (87.2–95.1)	0.090
Blastocysts	1.0 (0.7–1.3)^a^	3.9 (3.3–4.6)^b^	**<0.001**
Blastocyst rate (%)	9.9 (6.7–13.1)^a^	29.3 (25.0–33.6)^b^	**<0.001**
P4 (ng/mL)	6.3 (4.3–8.3)^a^	3.3 (1.5–5.0)^b^	**0.017**
E2 (pg/mL)	7.4 (6.0–8.8)	8.4 (7.6–9.3)	0.054
E2S (pg/mL)	75.5 (57.8–93.3)	114.1 (71.1–157.1)	0.074
AMH (pg/mL)	1,750.6 (1,197.9–2,303.3)^a^	2,556.6 (1,690.5–3,422.6)^b^	**0.043**
GDF9/BMP15 (AU)	263.9 (138.9–390.2)	1,239.7 (−615.7 to 3,095.9)	0.641
GDF9 (pg/mL)	54.3 (7.8–100.8); (*n* = 4)	1,363.2 (−2,849.7 to 5,576.0); (*n* = 4)	0.886
BMP15 (pg/mL)	79.6 (6.1–153.0); (*n* = 3)	214.7 (−220.9 to 650.3); (*n* = 4)	0.629

AU, arbitrary units; BCS, body condition score; P4, progesterone; E2, estradiol-17β; E2S, 17β-estradiol-3-sulfate; AMH, anti-Müllerian hormone; GDF9, growth differentiation factor 9; BMP15, bone morphogenetic protein 15.

**Figure 3:**
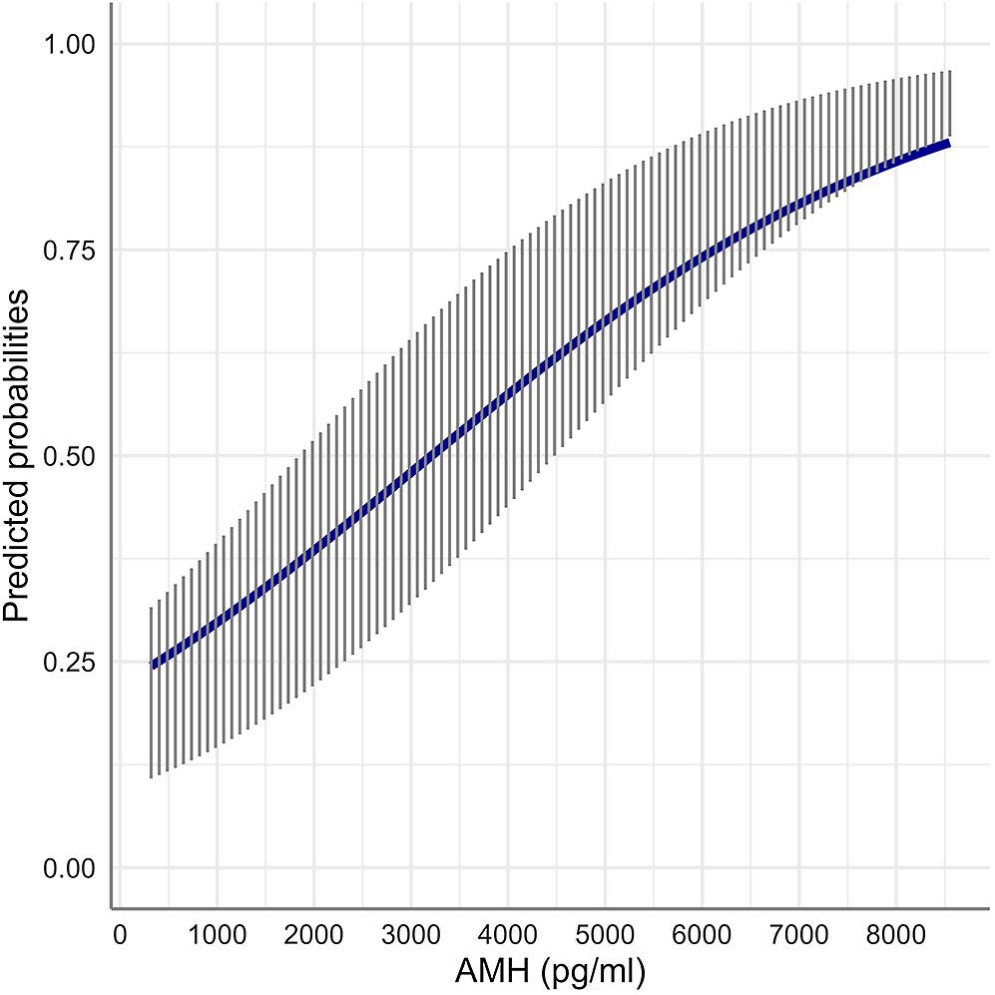
Average marginal probabilities (with the respective interquartile range) to obtain ≥3 embryos per OPU session (vs <3 embryos per OPU session) in relation to increasing serum AMH concentrations. Probabilities were computed after fitting generalized linear mixed models to 500 bootstrap samples of an original dataset of *n* = 59 mares and averaged across mares.

According to the generalized linear mixed model describing the relationship of the outcome of an OPU session (production of <3 vs ≥3 embryos per OPU session) with the mare’s hormonal profile, AMH concentration was the only deciding predictor of the dichotomous response variable. An increase in AMH concentration by 1,000 pg/mL (when all other predictors were kept constant) was linked to a 66% higher probability (odds ratio = 1.66, *P* = 0.028) of obtaining ≥3 embryos per OPU session (Fig. 3).

**Figure 4:**
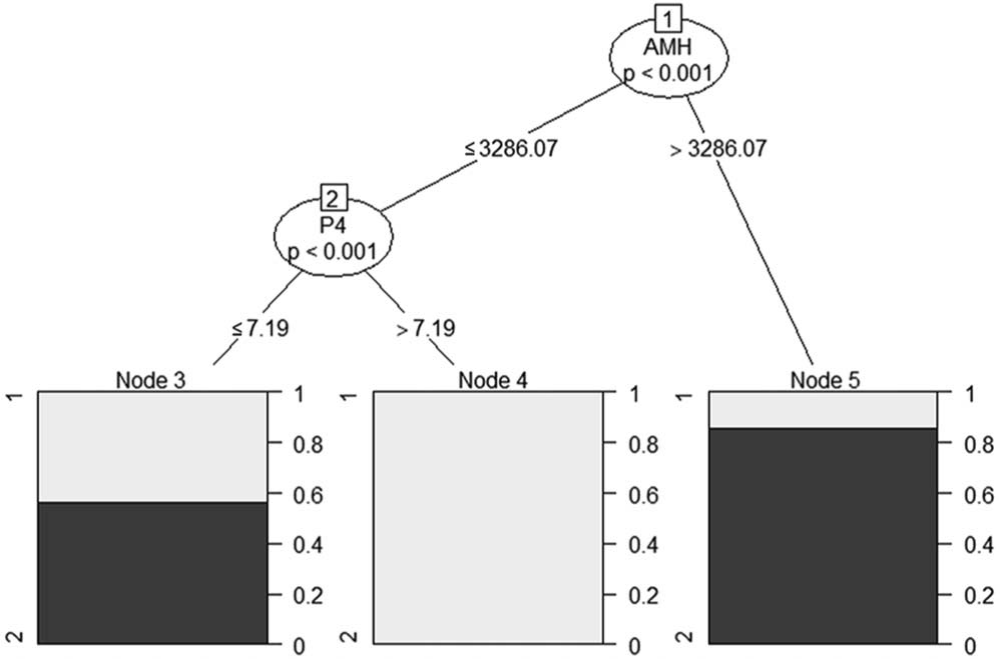
Example of RF tree with two continuous variables (AMH and P4) as predictors of the embryo yield (production of ≥3 embryos vs <3 embryos per OPU session). Concentrations of AMH and P4 were used as predictors in a tree with two-level depth. The cut-off value of the predictor is given along the branch (black line) originating from each decision node. Within each decision node, the predicted class probability *P* is also presented. At each terminal node, which no longer splits into sub-nodes, the ratio of mares producing ≥3 embryos (black area) or <3 embryos (gray area) is presented in a cumulative bar.

We also grew a forest for the classification of mares in the groups ‘≥3 embryos’ vs ‘<3 embryos’ per OPU session. In this case, AMH, along with P4, presented the highest MDA and MDGI and were, therefore, used as input for the RF. The RF showed an overall OOB error rate of 39%. Classification error rate for the assignment of mares in groups ‘<3 embryos’ and ‘≥3 embryos’ was 56 and 26.5%, respectively. Examples of a RF decision tree including AMH and P4 is demonstrated in Fig. 4. Overall, mares with AMH values >3,286.07 pg/mL had an 89% likelihood of yielding >3 embryos. For mares with an AMH concentration ≤3,286.07 pg/mL, P4 concentration was the deciding factor. In case of P4 concentration >7.19 ng/mL, the chance of producing ≥3 embryos was zero, whereas if P4 concentration ≤7.19 ng/mL, the mare still had a 58% chance of yielding ≥3 embryos per OPU session.

**Figure 5:**
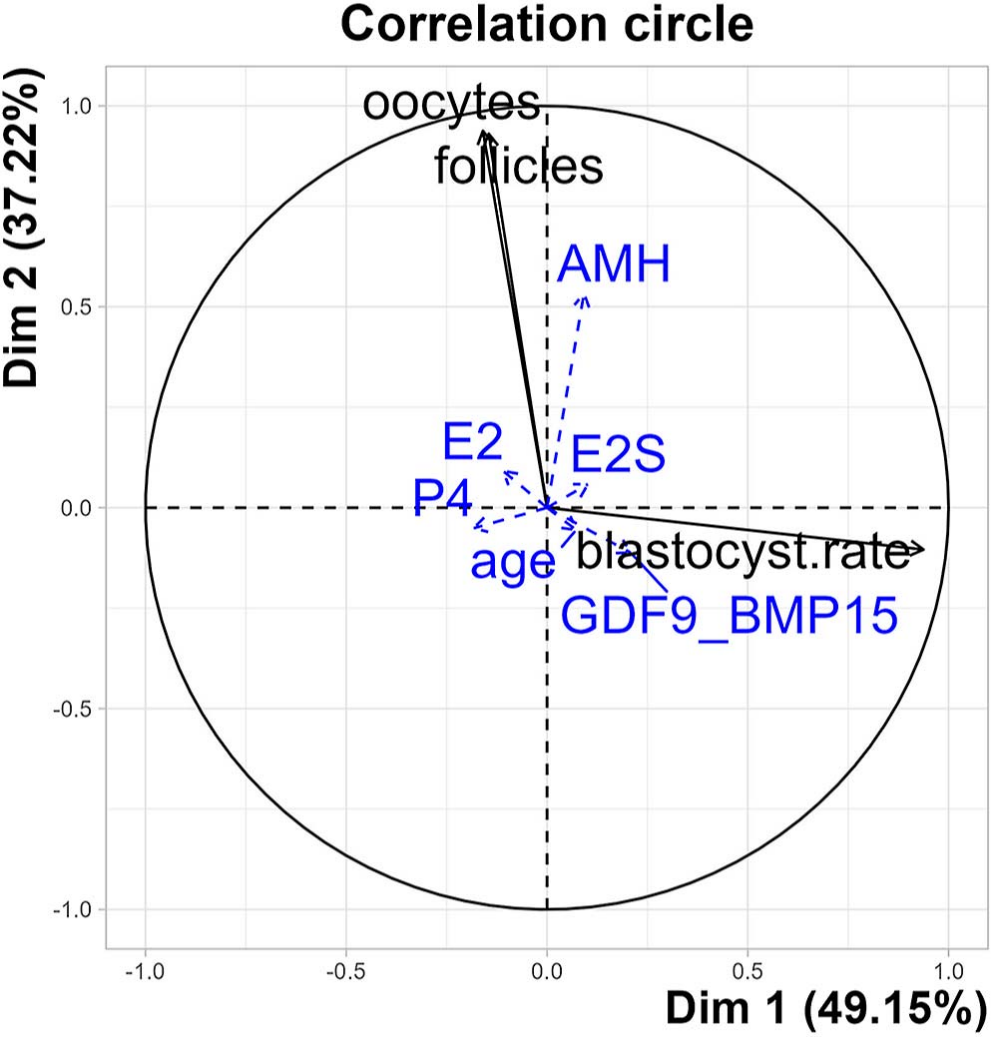
Principal component analysis variables correlation plot, showing the correlation of single continuous variables on the two principal components (dimension 1 and dimension 2) capturing 49.15 and 39.22% of the total variability of the dataset, respectively. Positively correlated variables are grouped together; negatively correlated variables are positioned in opposite quadrants. Variables presented with blue dashed lines were further evaluated as predictors for the assignment of mares into the embryo groups.

### Influence of individual mare characteristics on blastocyst rate

Since blastocyst rate was the only outcome differing between the low and high output groups, independent of the threshold set, we wanted to check if any of the other factors affected the blastocyst rate of each OPU session. As shown by principal component analysis (Fig. 5), GDF9/BMP15 complex concentrations and blastocyst rate clustered together, and both contributed to dimension 1. However, neither all-subsets regression nor linear regression showed a significant influence of hormonal traits, BCS, or age on the blastocyst rate (*P* > 0.05 in all cases).

## Discussion

This study aimed to analyze the suitability of the oocyte-secreted factors GDF9 and BMP15 as potential candidates for predicting OPU-ICSI outcome in horses. The serum concentrations of GDF9 and BMP15 were very low, with GDF9 being detected in 14% and BMP15 in 12% of all analyzed samples. This might be due to their origin in oocytes (Gilchrist *et al.* 2008). However, two mares had a GDF9 concentration 100-fold above the minimal detection limit of the assay and BMP15 concentrations 20-fold above, suggesting the existence of large variability between individuals. Nevertheless, neither of these parameters could be associated with OPU-ICSI outcomes or endocrine characteristics. While the more potent GDF9/BMP15 heterodimer was detected in all but two of the analyzed samples, it showed no difference between mares with low and high OPU-ICSI outcomes. Using serum AMH, P4, and E2 concentrations as input, RF trees were built to predict the probability of producing a minimum of two or three embryos per cycle, respectively. Our analyses suggest a 92% probability of producing at least two embryos per cycle if the P4 and E2 concentrations at the time of OPU are considered, supporting a possible association between estrous cycle stage and OPU-ICSI outcome in mares (Fonte *et al.* 2024). Moreover, lower progesterone and higher AMH concentrations were determined at the time of OPU in mares producing at least three embryos, strengthening the importance of estrous cycle stage but also of oocyte donor selection in obtaining above-average results.

Maternal age did not affect OPU-ICSI outcome in our study, possibly due to the limited number of cases. A decrease in the number of aspirated follicles and recovered oocytes is observed in mares older than 20 years (Claes *et al.* 2015, Cuervo-Arango *et al.* 2019*a*), and this can translate into a lower number of blastocysts produced per OPU session (Fonte *et al.* 2024). However, when selection of the mares based on antral follicle count is performed before OPU, neither the blastocyst rate nor the number of blastocysts per OPU-ICSI session is affected (Cuervo-Arango *et al.* 2019*a*, Papas *et al.* 2021). This study included eight mares older than 20 years, from which 1.6 embryos/OPU were produced on average. Besides maternal age, the breed effect on OPU-ICSI outcome is also well documented. For example, results obtained with Warmblood and American Quarter Horse are very similar (Fonte *et al.* 2024), whereas Arabians are well known for their poorer efficiency (Lazzari *et al.* 2020). Accounting for this aspect, only Warmblood and American Quarter Horse mares were included in this study, therefore the lack of a breed effect on the results was not surprising. Furthermore, each mare’s BCS at the time of oocyte collection was recorded and its relationship with OPU-ICSI outcome analyzed. In cattle, both under- and overnutrition are known to affect oocyte quality and *in vitro* embryo production (Ruiz *et al.* 1996, Armstrong *et al.* 2001), but similar information in horses is not yet available. Generally, it is desired that mares enter the breeding season at a BCS of 5.0 or above (Henneke *et al.* 1984), although obesity disturbs the equine ovarian follicle and alters the mitochondrial function of the oocyte (Catandi *et al.* 2024). This study included the first analysis, to our knowledge, of the effect of BCS on *in vitro* embryo development in horses and did not find any correlation. It is worth mentioning that the mares enrolled in our study had a BCS between 4.0 and 7.0 on a scale from 1.0 to 9.0, and this might have masked some effects expected in undernourished and obese mares.

Regardless of the embryo threshold used, the number of aspirated follicles, the number of oocytes collected, and the recovery rate did not differ between low and high outcome groups. Significant positive correlations were observed between the number of aspirated follicles and the number of oocytes collected, as well as the AMH levels. In mares, a greater total follicle number is observed when OPU is being performed during estrus, but there is no effect of the season (Choi *et al.* 2016, Walbornn *et al.* 2022, Fonte *et al.* 2024). Due to its relevance to the outcome of an OPU-ICSI session, and as demonstrated by the principal component analysis in our study showing a strong correlation between the number of follicles aspirated and oocytes recovered, the presence of at least ten follicles >5 mm is often a prerequisite for performing OPU in horses (Papas *et al.* 2021). As the recovery of oocytes is essential for a successful OPU-ICSI session (Cuervo-Arango *et al.* 2019*b*), we expected to see differences in the number of oocytes collected between mares with low and high outcomes, but this was obviously not the case. Intriguingly, although the number of oocytes recovered was very similar between the groups with different outcomes, independent of the threshold used, maturation and blastocyst rate strongly differed, suggesting that further individual factors are responsible for OPU-ICSI outcome.

Following the hypothesis that the identity of the mare represents the most reliable predictor for OPU-ICSI success (Cuervo-Arango *et al.* 2019*b*), serum AMH concentration was previously analyzed as a potential outcome predictor in horses due to its importance in folliculogenesis and its association with reproductive success in other mammals. Strong correlations have been observed between AMH levels and the number of oocytes recovered from young and middle-aged mares, but not from old mares (>16 years) (Papas *et al.* 2021). Indeed, AMH is a reliable biomarker for antral follicle count in mares of all ages (Claes *et al.* 2015, Scarlet *et al.* 2018), as it is only produced by the granulosa cells of antral follicles in horses (Ball *et al.* 2008). In cattle, despite being a reliable predictor of the ovarian response to superovulation (Rico *et al.* 2009), AMH is not associated with IVF success (Vernunft *et al.* 2015). In our study, we did not see any difference in AMH concentration between mares producing at least two embryos per OPU-ICSI session and those with lower outcome, but this changed when the threshold of three embryos was used. Specifically, higher AMH levels were measured in the serum of mares with an above-average OPU-ICSI outcome (≥3 embryos). This confirms the results of a previous study showing a higher number of blastocysts in mares with higher levels of AMH (Papas *et al.* 2021). Moreover, both studies demonstrate that AMH levels cannot be used as a 100% predictor for ICSI success in horses, despite using different AMH assays. With our setup, an AMH concentration >3,286.07 pg/mL would lead to the production of at least three embryos per OPU-ICSI session in 89% of the cases, which is worthy of the attention of clinicians and owners aiming at producing more embryos from a specific mare in a short time.

There is emerging information suggesting that the day of the estrous cycle upon which OPU is performed affects OPU-ICSI outcome in horses. Here, we observed a significantly lower P4 concentration in the group of mares with above-average results (≥3 embryos). Moreover, P4 concentration was indicated as a deciding factor in all RF trees created, independent of the number of embryos, and its high concentrations were associated with the production of fewer embryos. Strictly speaking, the ‘threshold values’ established in our study must be considered assay-specific, although our reference values for cyclic mares correspond quite precisely with the previously published mass spectrometric data (Conley *et al.* 2018). Despite the limited comparability of steroid hormone concentrations from different measurement methods, the P4 concentration, and thus the functional state of the ovary, clearly had an impact on the efficiency of the OPU-ICSI procedure in our study. In horses, peripheral P4 concentrations start increasing on the day of ovulation (Jacob *et al.* 2009) and reach their maximum 5 days later (Townson *et al.* 1989). This is followed by a plateau phase with concentrations above 10 ng/mL, and a drop below this value is first seen again 15 days after ovulation (Townson *et al.* 1989). While P4 continues to decrease, an increase in systemic E2 occurs on the day before follicle deviation (i.e. 15–16 days after ovulation), with peak levels 2 days before ovulation (Gastal *et al.* 1999, Ginther *et al.* 2006). Estradiol concentration at the time of oocyte collection is essential for further development, as oocytes recovered from immature follicles of mares exhibiting estrus are more likely to develop into a blastocyst compared with oocytes recovered from mares that do not exhibit estrus (Metcalfa *et al.* 2017). In this study, estradiol-17β concentrations were similar among mares with distinct OPU-ICSI outcome. However, once E2 concentrations rise beyond 11 pg/mL, a negative effect on the number of embryos produced is observed. Although it is not possible to accurately assign a single-point measurement of P4 and E2 to a specific day of the cycle, our data suggest scheduling oocyte collection during diestrus should be avoided in order to increase the chances of producing two or more blastocysts per OPU-ICSI session. Although the cycle stage did not directly affect the number of embryos produced, our observations suggest that high P4 concentrations are detrimental to equine embryo production. However, this needs to be evaluated with a larger dataset than the one used in this study, and the challenge of performing OPU in the presence of a dominant follicle due to the increased size of the ovary should also be considered.

Mares are known to produce significant amounts of sulfoconjugated estrogens in the preovulatory follicle (Brown *et al.* 2006), but systemic concentrations of estrone sulfate have only been determined in non-pregnant mares to date (Makawiti *et al.* 1983). Based on the interrelationship with other intrafollicular factors such as BMP15 (Samie *et al.* 2025), and considering that estrogen inactivation seems to be required for the oocyte to acquire developmental competence (Brown *et al.* 2004), here we determined 17β-estradiol-3-sulfate concentrations in relationship with OPU-ICSI outcome. We observed 10-fold higher concentrations of 17β-estradiol-3-sulfate in serum compared to the unconjugated form. In comparison to their unconjugated counterparts, 17β-estradiol-3-sulfate concentrations did not seem to be associated with any mare-related factors, nor with OPU-ICSI outcome, suggesting that changes in their intrafollicular concentrations might not necessarily be reflected in the serum levels.

The oocyte-secreted factors GDF9 and BMP15 have emerged as possible determinants of oocyte quality and quantity, based on their multiple roles in controlling ovarian function. In general, GDF9 and BMP15 act either as homodimers in synergistic cooperation or as biologically active heterodimers (Sanfins *et al.* 2018). The GDF9/BMP15 heterodimer is a potent regulator of granulosa cell function that improves embryonic development (Peng *et al.* 2013, Mottershead *et al.* 2015). Using different assays, GDF9 has been detected in the serum of up to 54% of women, whereas BMP15 detectability is slightly higher with up to 73% positive samples, confirming high interindividual variability (Riepsamen *et al.* 2021). Despite this, serum GDF9, but not BMP15, is positively correlated with antral follicle count and oocyte number, but not with AMH in women (Riepsamen *et al.* 2019, Riepsamen *et al.* 2023). We did not find any associations of these factors with other mare-related or OPU-ICSI outcome parameters in this study, presumably due to the low number of samples with detectable GDF9 and BMP15 concentrations and the relatively low number of mares included. Species-specific tests with higher sensitivity might be required to detect differences in serum concentrations of oocyte-secreted factors in horses. Although the GDF9/BMP15 heterodimer was detected in all but two of the samples, its levels were similar between the high and low outcome groups. Of all the parameters assessed, GDF9/BMP15 concentration was the only one to cluster together with blastocyst rate according to the principal component analysis. However, it cannot be used as a predictor for blastocyst rate according to regression analysis.

In conclusion, our results demonstrate differences between the progesterone and AMH serum levels in mares producing more than or fewer than three embryos per OPU-ICSI session. Moreover, our analysis suggests a possible relationship between progesterone and estradiol-17β concentrations at the time of OPU and the number of embryos produced by ICSI. Despite not being associated with embryonic development in this study, GDF9 and BMP15 are worth further investigation with more sensitive assays and with a larger sample size. Regardless of the embryo number used as the threshold, high progesterone concentration was associated with a poorer outcome in this study. Further studies should analyze the association between oocyte collection on specific days during estrus and OPU-ICSI outcome in horses.

## Declaration of interest

The authors declare that there is no conflict of interest that could be perceived as prejudicing the impartiality of the work reported.

## Funding

This research did not receive any specific grant from any funding agency in the public, commercial, or not-for-profit sector.

## Author contribution statement

DS conceived the study, interpreted data, and wrote the manuscript. GS, SC, GL, and CG acquired data. EM analyzed data. HB provided resources, supervised the study. MPK provided resources and supervised the study, performed knowledge transfer, and was involved in interpretation of data, drafting, and editing the manuscript. The manuscript was revised by all authors.
